# Dysplastic Nodules with Glypican-3 Positive Immunostaining: A Risk for Early Hepatocellular Carcinoma

**DOI:** 10.1371/journal.pone.0087120

**Published:** 2014-01-31

**Authors:** Li Gong, Long-Xiao Wei, Pin Ren, Wen-Dong Zhang, Xiao-Yan Liu, Xiu-Juan Han, Li Yao, Shao-Jun Zhu, Miao Lan, Yan-Hong Li, Wei Zhang

**Affiliations:** 1 The Helmholtz Sino-German Laboratory for Cancer Research, Department of Pathology, Tangdu Hospital, The Fourth Military Medical University, Xi’an, P.R China; 2 Department of Nuclear Medicine, Tangdu Hospital, The Fourth Military Medical University, Xi’an, P.R China; 3 Department of Gynaecology and Obstetrics, Tangdu Hospital, The Fourth Military Medical University, Xi’an, P.R China; University of Pisa, Italy

## Abstract

Glypican-3 (GPC3) has been reported to be a novel serum and histochemical marker for HCC. The positivity or negativity for GPC3 in hepatic precancerous lesions, such as dysplastic nodules (DN), has also been described. Moreover, our previous studies have demonstrated that some DN in liver cirrhosis represent monoclonal hyperplasia, and confirmed their neoplastic nature. However, additional studies must be performed to investigate further the relationship between DN with GPC3 positivity and HCC. Thus, we first investigated the expression of GPC3 in 136 HCC and 103 small DN (less than 1 cm in diameter) by immunohistochemical staining and determined the clonality of 81 DN from female patients using X-chromosome inactivation mosaicism and polymorphism of androgen receptor (AR) gene. Then we examined these samples for chromosomal loss of heterozygosity (LOH) at 11 microsatellite polymorphism sites. The results demonstrated that GPC3 immunoreactivity was detected in 103 of 136 HCC (75.7%) and 19 of 103 DN (18.4%), and the positive ratio correlated with HBsAg positivity. Clonality assays showed that 15 GPC3-positive DN from female patients, including 12 high-grade DN (HGDN), and 28 (42.4%) of 66 GPC3-negative DN, were monoclonal. In addition, among 19 GPC3-positive DN, chromosomal LOH was found at loci D6S1008 (100%, 19/19), D8S262 (52.6%, 10/19) and D11S1301 (57.9%, 11/19). However, the LOH frequency in GPC3-negative DN was 5.95% (5/84), 23.8% (20/84), and 4.76% (4/84) in three loci, respectively. Thus, we concluded that GPC3-positive DN, especially GPC3-positive HGDN, was really a late premalignant lesion of HCC.

## Introduction

Hepatocellular carcinoma (HCC) is the fifth most common cancer and the third most common cause of cancer-related deaths worldwide [1]. Up to 80% of HCCs develop in the setting of liver cirrhosis related to hepatitis B and C virus infections. Different lesions have been suggested to represent preneoplastic conditions in human liver. They include dysplastic nodules (DNs) and dysplastic foci (DF), which are further classified into large cell change (LCC) and small cell change (SCC) [2], [3]. Whether both of these types of lesions represent true precursor lesions and which of these precursors is more closely related to HCC are intensely debated. Mounting evidence suggests that SCC represents precursor lesions that are more advanced than LCC in the course of human hepatocarcinogenesis [4]. Both lesions contain shorter telomeres compared with surrounding non-dysplastic hepatocytes of cirrhosis, but only SCC shows a high prevalence of checkpoint abrogation. The above data support the preneoplastic status of SCC in human hepatocarcinogenesis.

DNs, including low grade dysplastic nodular (LGDN) and high grade dysplastic nodular (HGDN), are distinctly nodular lesion that differ from the surrounding liver parenchyma regarding size, color, and texture. They are usually detected in cirrhotic livers, and most of them measure about 1 cm in diameter. Histologically, LGDN shows mild cytologic atypia and increase in cell density with a monotonous pattern compared with that of cirrhotic nodules. LCC may be found, but SCC is not present. HGDN always shows a certain degree of cytological and architectural atypia but insufficient for a diagnosis of malignancy. SCC is frequently seen inside the nodule but LCC can also be detected. The premalignant nature of DNs has been supported by some evidences [5].

Regarding the occurrence of preneoplastic and neoplastic nodules during hepatocarcinogenesis as described by Park [5], these precancerous lesions develop gradually into early HCC which corresponds to in situ or microinvasive carcinoma; and then develop into progressive HCC through the stage of “nodule-in-nodule”-type HCC. In this line, we propose that surveillance of at-risk cirrhotic population with special focus to their genetic changes could aid in the earlier detection of HCC and may decrease its morbidity and mortality rates.

Glypican-3 (GPC3) has been reported as a novel serum and histochemical marker for HCC by several groups [6], [7], [8]. It is an oncofetal protein and expressed abundantly in the fetal liver. It is also inactive in the normal adult liver, and shows great promise as an adjunct in the diagnosis of HCC. Positive immunostaining for GPC3 has been reported in 52.5% to 85% of HCCs [9], [10], [11], [12], [13], [14], [15], [16], [17], [18]. Simultaneously, the positivity for GPC3 of DN has been reported [19], [20]. However, it is not clear whether a few GPC3-positive nodules are indicative of HCC and their molecular genetic changes.

Monoclonality is a major characteristic of most tumors. By contrast, normal tissue and reactive hyperplasia are polyclonal. A lesion, which is neoplasm or reactive hyperplasia, may be determined by clonality assay based on X-chromosome inactivation mosaicism and polymorphism at the phosphoglycerate kinase (PGK) and androgen receptor (AR) loci in female somatic cells. In the previous studies, we ever examined the clonality of some small DN (less than 1 cm in diameter) in liver cirrhosis, and the results demonstrated that part of LGDN and all HGDN in liver cirrhosis tissue represented monoclonal hyperplasia. The occurrence of HGDN with SCC is a late event during DN progression and considered to be a premalignant morphologic phenotype [21]. These observations further support the contention that DN is a premalignant lesion of HCC.

In order to assess further the relationship between DN with GPC3 immunostaining and HCC, we investigated the expression of GPC3 in 136 cases of HCCs and 103 DN (including 30 HGDN and 73 LGDN), and examined the clonality of DN from female patients using X chromosome inactivation mosaicism and polymorphism of androgen receptor (AR) genes in female somatic cells, and observed the chromosomal loss of heterozygosity (LOH) of all DN at 11 microsatellite loci.

## Materials and Methods

### 2.1. Ethics Statement

The study protocol was approved by the Medical Ethics Commission of the Fourth Military Medical University in Xi’an, China. Written informed consent from all participants involved in our study was obtained.

### 2.2. Samples

Liver tissue samples from 136 surgically resected HCC, consisting of 128 cases with and 8 cases without clinical data, were collected between January 2007 and December 2011 from Tangdu Hospital, the Fourth Military Medical University (Xi’an, China). Each case was examined by three pathologists and diagnosed according to the World Health Organization International Histological Classification of Tumors. HCC samples were graded according to Edmondson’s criteria. All samples were surgically resected, fixed in 10 g/L neutral formalin, and embedded in paraffin. Serial sections were stained with hematoxylin and eosin (H/E). 103 small DN (less than 1 cm in diameter), including 81 from females and 22 from males, was classified according to the criteria of the International Working Party. Specifically, the morphological characteristics of LGDN are same as that of so-called regenerative nodules. They usually show mild increase in cell density with a monotonous pattern and/or clonal changes composed of glycogen-storing clear cells and glycogen-depleted amphophilic cells. LGDN may show bland cytologic but not frank architectural atypia. LCC is frequently seen inside and outside the nodules as microscopic dysplastic foci. HGDN is composed of glycogen-storing clear cells and basophilic cells, and shows an increased cell density, a certain degree of cytological and architectural atypia, but insufficient for a diagnosis of malignancy. SCC is frequently seen inside the nodule.

### 2.3. Immunohistochemistry

Sections (4-µm thick) from a representative block from each case were deparaffinized, rehydrated in graded alcohols, incubated with H_2_O_2_ to block the activity of endogenous peroxidases; and then subjected to heat-induced epitope retrieval in 0.1 mol/L citrate buffer at pH 6.0 in a microwave for 20 minutes. The slides were then incubated with a primary monoclonal antibody specific for GPC3 (dilution, 1∶200, Maixin Ltd Company, Fuzhou, China) for 2 hours at room temperature. After incubation with a rabbit anti-mouse secondary antibody, a subsequent reaction was performed using a biotin-free horse-radish peroxidase enzyme-labeled polymer and visualized using the EnVision plus detection system. The chromogen 3,3′-diaminobenzidine (Dako) was used and sections were counterstained with hematoxylin. Placental tissues were used as positive controls. Nonspecific IgG was used as a negative control.

The GPC3 staining was considered positive when the granular brown reaction was found in the cytoplasm and/or the membranes. Therefore, each case was observed by three pathologists. In detail, immunoreactivity was determined semi-quantitatively by examining fields (magnification, ×200). Using a (0–3+) scale, the staining was described as 0 staining (negative), 1+ staining (<10% of cells), 2+staining (10%–25% of cells), or 3+staining (>25% of cells). Statistical analysis was performed using the 2-tailed Fisher exact test or the χ^2^ test with the Yates continuity correction. A *P* value of <0.05 was considered statistically significant.

### 2.4. Laser Microdissection and DNA Extraction

We selected the corresponding paraffin block with DN, specially GPC3-positive DN, according to the results of immunohistochemical staining, and then prepared eight serial 10-µm tissue sections which were then placed on a UV-absorbing membrane for laser microdissection by LMD6000 (Leica Microsystems Ltd, Wetzlar, Germany). After HE staining, the slides were mounted on a microstat, and the selected nodules were dissected by a UV laser through motorized optical beam scanning. The dissected tissues (with the attached specimen ) were pooled into the cap of a 0.5-ml microcentrifuge tube that was filled with 40 µL lysate buffer and 10 µL protease K. Along with each dissected nodule, the surrounding normal liver tissue of the same size was isolated and analyzed as a control. The microcentrifuge tubes were placed in a waterbath (48°C) to digest the tissue specimens. After digestion for 12–20 h, genomic DNA was extracted using Qiagen blood & tissue Kit (Germany) and confirmed by gel electrophoresis (20 g/L agarose ), and then stored at −20°C until use.

### 2.5. Polymerase Chain Reaction (PCR) Amplification for Clonality

Nested polymerase chain reaction (PCR) was used to amplify the length polymorphism of CAG short-tandem repeat (STR) in exon 1 of the AR gene loci as described previously [22]. The genomic DNA extracted from DN and the surrounding normal liver tissues for the AR gene 10 µL each, was incubated with 5 U of *Hha* I (Promega, Madison, WI, USA) at 37°C for 3 h in a volume of 20 µL containing 2 µL of 10× reaction buffer. The digested DNA samples, 1 µL each, were then subjected to nested PCR. The reaction mixture was 50µL in volume, containing 4 µL of 10 mM dNTP (Gibco BRL, Life Technologies, Inc., Gaithersburg, MD, USA), primers AR1A and AR1B (20 µM each), and 5 µL of 10× buffer, 1.5 µL of 50 mM MgCl_2_ and 2.5U of Taq DNA polymerase (Gibco BRL). The amplification was conducted using a PT-200 thermocycler (MJ Research, Inc., Watertown, MA, USA) for 25 cycles (94°C for 40 sec, 56°C for 50 sec, and 72°C for 1 min) for the first round. PCR products (1 µL) were used as templates for a second PCR reaction using primers AR2A and AR2B. The amplification procedure was the same as that in the first round. Finally, the amplification efficiency was checked by resolving PCR reaction aliquots on 2% agarose gels. The PCR products, 4 uL for each, were also mixed with the same volume of loading buffer (1 g/L xylene cyanole, 1 g/L bromophenol blue, in formamide), and resolved on an 8% polyacrylamide gel containing 8 mol/L urea using the Mini-VE system (Amersham Biosciences, San Francisco, CA, USA) at 120 V for 4 h. Bands were visualized using silver staining.

### 2.6. Analysis and Assessment of PCR Products

Images of PCR gels were recorded and the intensities of the PCR bands for both alleles were quantified using an image-analyzing system (LabWorks 3.0, UVP, Cambridge, UK). A reduction in fluorescence intensity of at least 50% for either band, as compared to the intensity of the bands obtained in the absence of *Hha* I digestion, was used as an indicator of a loss of X chromosome inactivation mosaicism [22]. A corrected ratio (CR) was calculated by dividing the ratio of the upper-band intensity to the lower-band intensity, or vice-versa, of the same sample before and after digestion to give a CR value >1. In the present study, a CR value ≥2 was used to define a loss of X chromosome inactivation mosaicism.

### 2.7. LOH Analysis

The DN and the surrounding normal liver tissues were analyzed for LOH by PCR amplification of polymorphic microsatellite markers. Eleven microsatellite markers (Table 1) were selected according to our previous data of LOH loci that occur at high frequency in HCC. For PCR amplification, 1 µL DNA samples were subjected to PCR. The reaction mixture was 50µL in volume and contained 1.25 µL of 10 mM dNTP (Gibco BRL, Life Technologies, Inc., Gaithersburg, MD, USA), primers 1A and 1B (20 µM each), and 5 µL of 10× buffer, and 2.5U of Taq DNA polymerase (Gibco BRL). The amplification was conducted using a PT-200 thermocycler (MJ Research, Inc., Watertown, MA, USA) for 35 cycles or 25 cycles (95°C for 40 sec, 60°C for 50 sec, and 72°C for 1 min).

**Table 1:** Microsatellite markers used in this study.

No.	Locus	Marker	Forward	Reverse	PCRproductsize (bp)
1	16q23.3-q24.1	D16S518	**GGCCTTTTGGCAGTCA**	**ACCTTGGCCTCCCACC**	271–290
2	16q23.3-q24.1	D16S3049	**GCAATGAAGGCAACAAAGT**	**TTAAAAGACCTGGGGGAAT**	233–255
3	16q23.3-q24.1	D16S3096	**GATCTGGCTTACGATGATTTCTAAC**	**CCGTGATGATGTCTGCAAC**	173–219
4	16q23.3-q24.1	D16S3029	**ATAGAGTTGGGCTGCATAGA**	**CTTTCCTGAAATTGGAAGTGA**	271–299
5	16q23.3-q24.1	D16S504	**AGCTTGTTCAGGGAAACC**	**CAGGGATGTAGGACGTAGG**	149
6	16q23.3-q24.1	D16S684	**GGCCAAAAAAGCAGATTGG**	**TGGAATTTGAGTGGCTTTCT**	337
7	6q26	D6S305	**CACCAGCGTTAGAGACTGC**	**GCAAATGGAGCATGTCACT**	204–230
8	6q23-q25	D6S1035	**ACTTGAATCCAGGCATTCAG**	**AAAACTCAAGCTCAGAAAGGC**	130–152
9	6q26	D6S1008	**AAGAAAGACTAGAGAGACAGACAGC**	**ATCATTTGCCCATTTACCAA**	246–267
10	11p13	D11S1301	**GGCAACAGAGTGAGACTCA**	**GTGTTCTTTATGTGTAGTTC**	336
11	8p23.2	D8S262	**AGCTCAAAAGCGAAGGTGAT**	**GGCAACAAAGTGAGATCCTG**	114–128

Images of PCR gels were recorded and the intensities of the PCR bands for both alleles were quantified using an image-analyzing system (LabWorks 3.0, UVP, Cambridge, UK).

A reduction in fluorescence intensity by 50% or greater in 1 or more allele in the DN as compared to an identical allele in a normal tissue was considered an indicator of LOH. Statistical analysis was performed using the 2-tailed Fisher exact test with the Yates continuity correction. A *P* value of <0.05 was considered statistically significant.

## Results

### 3.1. Clinicopathologic Features of HCC

Of the 128 patients with clinical data, there were 108 males and 20 females, with a male-to-female ratio of 5.4∶1. There were 82 (75.9%) males and 17 (85%) females with liver cirrhosis. The ages of patients with HCC ranged from 27 to 84 years (mean: 52 years). Unfortunately, no other information was obtained in 26 out of 128 patients except for age, sex, and HCC grade. Hepatitis B surface antigen (HBsAg) and hepatitis C virus (HCV) were detected in the sera of 61.76% (63/102) and 5.88% (6/102) patients, respectively. 56.86% (58/102) of patients had elevated serum alpha-fetoprotein (AFP) levels with values of 21.51–35350 ng/mL. Histopathologically, 10.94% (14/128) of HCC were well-differentiated, 60.93% (78/128) were moderately differentiated, and 28.13% (36/128) were poorly differentiated (Table 2).

**Table 2:** Relationship between glypican-3 (GPC3) expression and clinical features of patients with hepatocellular carcinoma.

Wk13374861variables	GPC3-positive	GPC3-negative	χ^2^	*P*
Age (yrs)				
≤52 (n = 68)	51	17	0.046	0.842
>52 (n = 60)	44	16		
Sex				
Male (n = 108)	81	27	0.22	0.781
Female (n = 20)	14	6		
AFP				
≥20 ng/mL (n = 58)	46	12	0.061	0.813
<20 ng/mL (n = 44 )	34	10		
HBsAg or HCV				
+ (n = 69)	62	7	16.453	0.000
− (n = 33)	18	15		
HCC differentiation				
Grade I (n = 14)	8	6	2.443	0.295
Grade II (n = 78)	60	18		
Grade III (n = 36)	27	9		
Tumor size				
≥5 cm (n = 47)	37	10	0.004	0.947
<5 cm (n = 55)	43	12		
Extrahepatic metastasis				
Presence (n = 32)	25	7	0.003	0.959
Absence (n = 70)	55	15		

### 3.2. GPC3 Expression in HCC and DN

**Figure 1:**
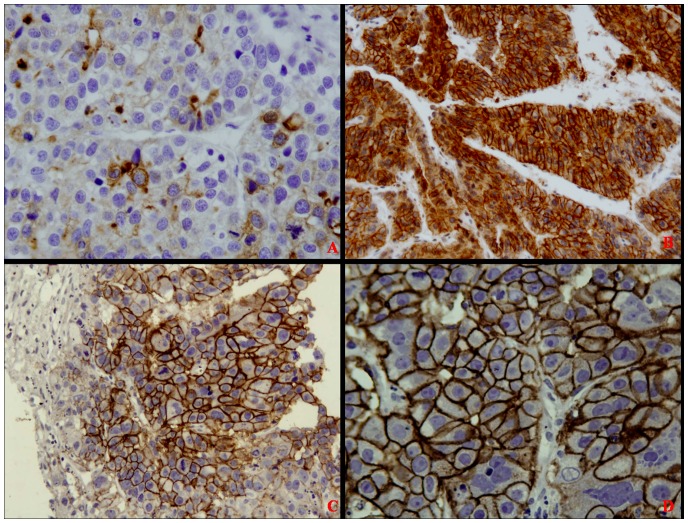
GPC3 immunohistochemical staining in HCC. (Figure A ×200, B ×200, Figure C ×200, D ×400). A) weakly positive; B, C, and D) strongly positive.

We assayed for GPC3 expression in 136 cases of HCC, and found that 103 cases (75.7%) stained positive for GPC3. The amount of reactivity was graded as 1+ (<10% of cells) in 13 cases (12.62%), 2+ (10%–25% of cells) in 22 cases (21.36%), and 3+ (>25% of cells) in 68 cases (66.02%). Next, we evaluated the expression of GPC3 in relation to the clinicopathological features, such as age, sex, AFP level and HBsAg or HCV in serum, HCC differentiation, tumor size, and metastasis. GPC3 expression closely correlated with HBsAg positivity (*P*<0.05). Although GPC3 expression was higher in moderately and poorly differentiated HCC than in well differentiated HCC, this difference in expression was not statistically significant (*P* = 0.295). Similarly, the difference in GPC3 expression in HCCs with or without extrahepatic metastasis did not reach statistical significance (*P* = 0.959) (Table 2). Moreover, we found 3 different immunostaining patterns in GPC3 positivity for HCC: predominantly cytoplasm (Figure1A), predominantly membrane (Figure1C, D), and both membrane and cytoplasm (Figure1B). However, these 3 different staining patterns did not appear to correlate with the differentiation status of HCC, ages, and gender.

**Figure 2:**
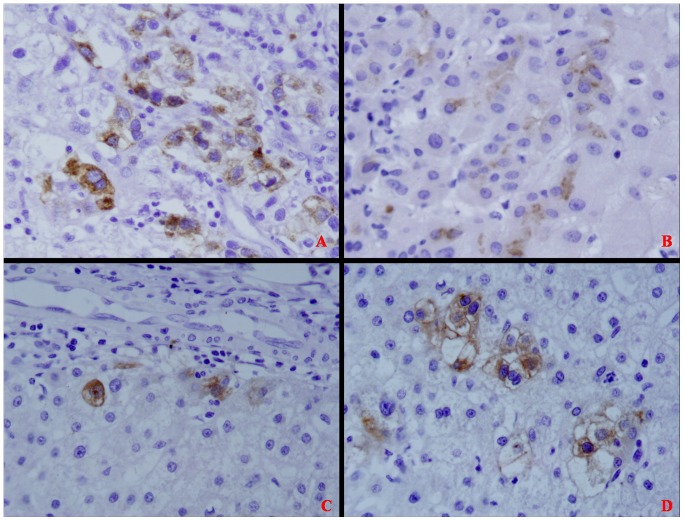
Weak and focal immunohistochemical staining of GPC3 in several different DN. (A ×400; B ×400; C ×400; D ×400).

**Figure 3:**
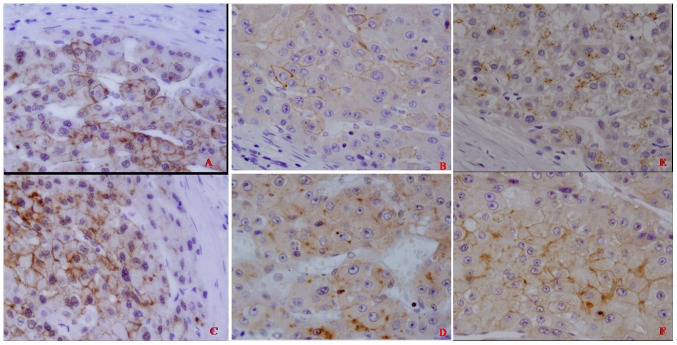
Widespread immunohistochemical staining of GPC3 in five different DN. (A ×400; B ×400; C ×400; D ×400; E ×400; F ×400).

GPC3 staining was performed in 103 DN, including 30 HGDN and 73 LGDN. The results demonstrated that 19 DN (18.4%), including 15 HGDN and 4 LGDN, were positive (Table 3). The ratio of HGDN (15/30, 50%) was obviously higher than that of LGDN (4/73, 5.48%), and this difference is statistically significant (P<0.05) (Table 4, 5). The amount of reactivity was graded as 1+ (<10% of cells) in 4 cases (21.05%), 2+ (10%–25% of cells) in 10 cases (52.63%), and 3+ (>25% of cells) in 5 cases (26.32%). Moreover, we noticed that there were two types of GPC3 expression in DN. Only several hepatocytes were positive for GPC3 in some DN (Figure2), but the whole nodule was positive for GPC3 in the other DN (Figure3). The positive immunostaining pattern of GPC3 for DN was similar to that in HCC. Namely, the positive reactivity for GPC3 was predominantly in membrane (Figure3A, 3B, 3C, and 3F) or cytoplasm (Figure3D, and 3E).

**Table 3:** Clinical information of the 20 female and 9 male patients enrolled in this study and the pathological features of their liver lesions.

Case(No)	Age	HBsAg	AFP(ug/l)	Pathological diagnosis andHCC grade	Number of DN(HGDN)	Number of monoclonal lesions(GPC3 positive DN)
01	59	+	350	HCC III, liver cirrhosis	4 (1)	1
02	64	+	68.25	HCC II, liver cirrhosis	4 (1)	1
03	42	+	236.3	HCC III, liver cirrhosis	6 (2)	3 (2)
04	79	+	1730	HCC II-III, liver cirrhosis	8 (3)	5 (2)
05	57	+	28.45	HCC II, liver cirrhosis	3	1
06	50	+	124	HCC I, liver cirrhosis	4 (1)	2
07	51	+	44.51	HCC I-II, liver cirrhosis	6 (2)	3 (1)
08	33	−	48377	HCC II-III	0	
09	56	+	22.99	HCC II, liver cirrhosis	5 (1)	3 (1)
10	59	+	11746	HCC II, liver cirrhosis	10 (4)	6 (3)
11	57	+	496.1	HCC II, liver cirrhosis	4 (1)	3
12	48	−	1429	HCC III	0	
13	54	+	28.50	HCC II-III, liver cirrhosis	4	2 (2)
14	39	+	23631	HCC II, liver cirrhosis	8 (4)	6 (2)
15	41	+	34.48	HCC I-II, liver cirrhosis	3	1 (1)
16	32	+	53109	HCC II, liver cirrhosis	3 (1)	1
17	76	−	18.6	HCC II	0	
18	40	+	65.99	HCC II-III, liver cirrhosis	6 (2)	4 (1)
19	39	+	505.3	HCC II-III	0	
20	49	−	17.5	HCC II-III, liver cirrhosis	3 (1)	1
21	38	−	350	HCC II, liver cirrhosis	2	
22	58	+	35350	HCC II, liver cirrhosis	3 (1)	(1)
23	63	+	104.61	HCC II, liver cirrhosis	3 (1)	(0)
24	49	+	7.37	HCC I, liver cirrhosis	2 (1)	(1)
25	35	+	4761	HCC III, liver cirrhosis	2 (2)	(2)
26	34	+	350	HCC III, liver cirrhosis	2 (1)	
27	44	+	2344	HCC II, liver cirrhosis	3	
28	68	+	157	HCC III, liver cirrhosis	3	
29	71	−	7.95	HCC I, liver cirrhosis	2	

**Note:**case 1–20, female patients; case 21–29, male patients.

**Table 4:** The details of 81 DN from females with GPC3 and clonality.

		LGDN (n = 57)		HGDN (n = 24)	
		GPC3		GPC3	
		positive	negative	positive	negative
**Monoclonal**	positive	3	16	12	12
	negative	0	38	0	0

**Table 5:** The expression condition of GPC3 in 22 DN from males.

		LGDN (n = 16)	HGDN (n = 6)
**GPC3**	positive	0	4
	negative	16	2

### 3.3. GPC3 Expression in HCC and its Adjacent DN

In the same patient, we found that GPC3 expression in HCC to be congruent with the adjacent DN. In other words, GPC3 was detected in the adjacent HCC if it was also detected in the DN (Figure2, and Figure3). On the contrary, it was not necessary to express GPC3 in the adjacent DN if it was positive for HCC. Thus, we concluded that GPC3-positive DN was closely related to HCC.

### 3.4. Clonality Determination of DN

**Figure 4:**
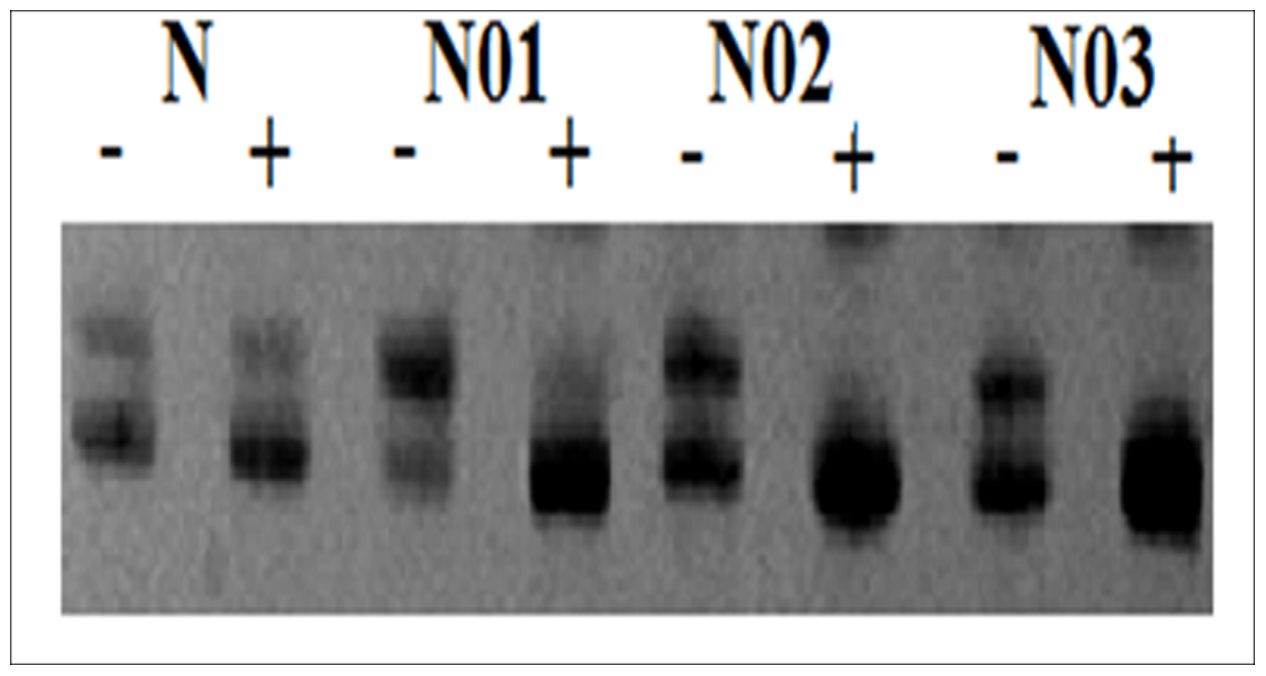
Clonality analysis revealed that one band disappeared when DN was digested with *Hha* I, but two bands remained when the surrounding liver tissue was digested with *Hha* I. N, the surrounding liver tissue; N01-N03, 3 GPC3-positive staining DN; −, without *Hha* I digestion; +, with *Hha* I digestion.

The clonality assay was used to detect the nature of 81 DN from females at the AR loci. In these samples, 15 GPC3-positive DN (including 12 HGDN and 3 LGDN) and 28 (12 HGDN and 16 LGDN) out of 66 GPC3-negative DN showed loss of X-chromosomal inactivation mosaicism upon digestion with *Hha* I (Table 3 and Table 4). In other words, all of HGDN (100%, 24/24) and 33.3% (19/57) LGDN were monoclonal. The results further confirmed our previous observations [21]. The AR PCR gel pictures showed that the DNA samples obtained from tissues without *Hha* I digestion produce two bands. When the DNA samples from DN with GPC3-positive reactivity were digested with *Hha* I, one of the two bands disappeared, indicating neoplastic hyperplasia. However, the surrounding normal liver tissue digested with *Hha* I still showed two bands with equal intensities (Figure4). In addition, we found that 3 GPC3-positive LGDN with monoclonal hyperplasia were from two patients without HGDN (case 13 and case 15, Table 3). Moreover, the 3 GPC3-positive LGDN located in adjacent HCC. This indicated that part of GPC3-positive LGDN might directly progress into HCC.

### 3.5. LOH Analysis

**Figure 5:**
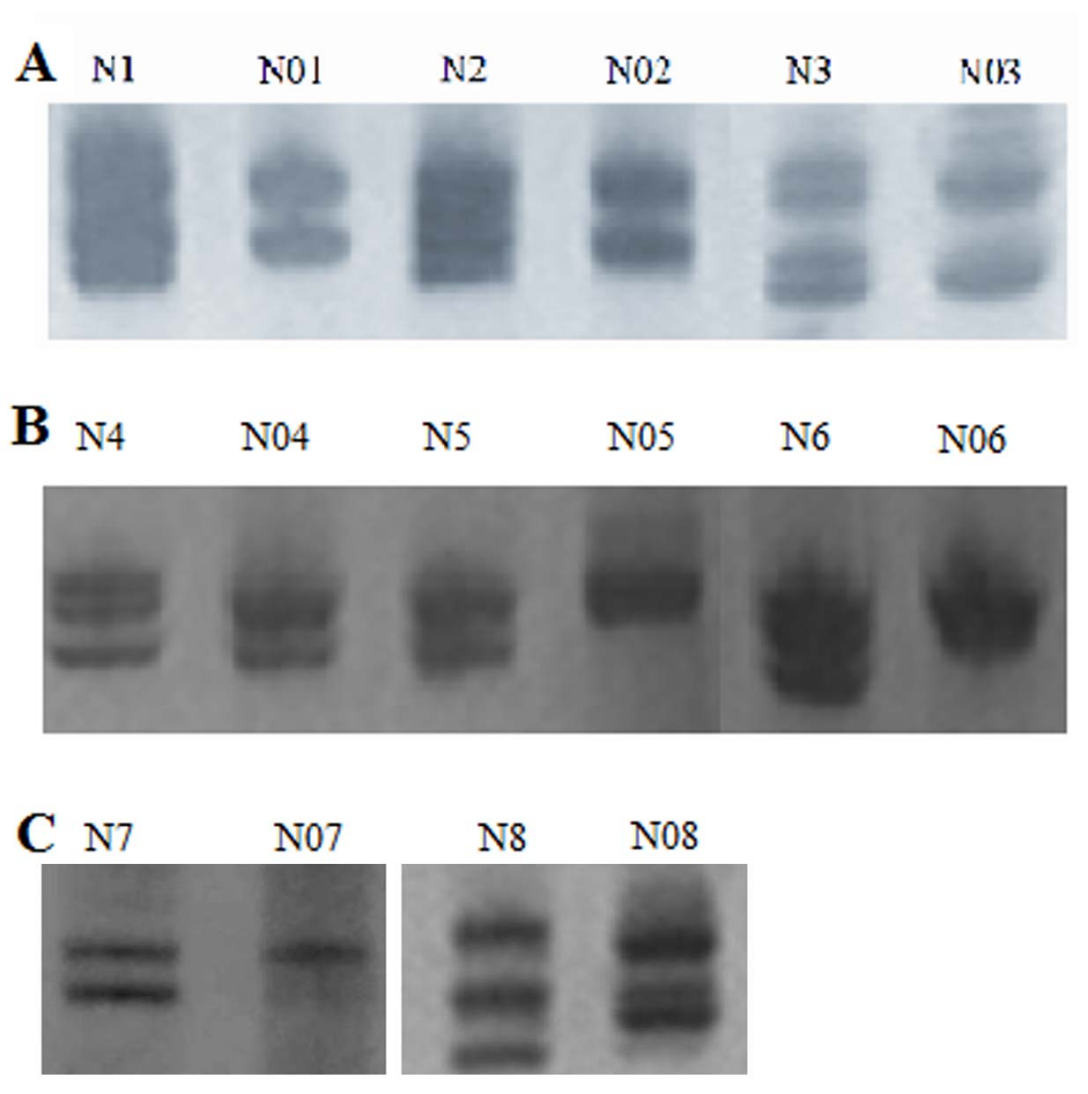
The result of LOH analysis at D8S262 (A), D11S1301 (B) and D6S1008 (C): A) one or two bands from monoclonal GPC3-positive DN disappeared. These bands are present in normal liver tissues. N1-N8, normal liver tissue; N01-N08, 8 different monoclonal GPC3-positive DN.

LOH was detected at the loci D8S262, D11S1301 and D6S1008, but not at other microsatellite markers. In the 19 GPC3-positive DN, chromosomal LOH was found at loci D8S262 (52.6%, 10/19), D11S1301 (57.9%, 11/19) and D6S1008 (100%, 19/19) (Table 6, Figure 5). However, the frequency of LOH in GPC3-negative DN was 23.8% (20/84), 4.76% (4/84), and 5.95% (5/84), in the above three loci, respectively. Namely, Fisher’s exact test demonstrated that the frequency of LOH was differentially higher in GPC3-positive DN compared to GPC3-negative DN at the above three loci (Table 6). Moreover, this difference is also statistically significant (P<0.05). In addition, the ratio of HGDN was higher than LGDN once there was chromosomal LOH at any loci. This indicated that there was a close relationship between HGDN and its adjacent HCC.

**Table 6:** The LOH frequency of GPC3-positive and GPC3-negative DN at three loci.

DN	n	D8S262	D11S1301	D6S1008
		% (HGDN+LGDN)/n	% (HGDN+LGDN)/n	% (HGDN+LGDN)/n
**GPC3-positive DN**	19	52.6% (10+0)/19	57.9% (11+0)/19	100% (15+4)/19
**GPC3-negative DN**	84	23.8% (14+6)/84	4.76% (4+0)/84	5.95% (5+0)/84
Chi-square		6.236	35.158	76.688
*P*		0.013	<0.001*	<0.001*

Fisher’s exact test demonstrated that the frequency of LOH was differentially higher in GPC3-positive DN compared to GPC3-negative DN at the above three loci.

## Discussion

Glypican-3 (GPC3) is an oncofetal antigen and a member of the glypican family of the membrane-bound heparan sulfate proteoglycans. It has roles in the development and regulation of cellular proliferation and apoptosis in specific tissues [23], [24]. The gene is expressed in fetal liver and progenitor cells, as well as in many cases of HCC and in other tissues. Recently, several studies have demonstrated that GPC3 is positive in most HCC, and positive immunostaining has been detected in 52.5% to 85% of HCC, but not in healthy livers or various benign liver lesions, including cirrhotic livers, hepatocellular adenoma and focal nodular hyperplasia. This pattern of expression suggests great promise for GPC3 as a diagnostic marker of HCC [9], [10], [11], [12], [13], [14], [15], [16], [17], [18]. In our studies, GPC3 immunoreactivity was detected in 103 of 136 HCC cases (75.7%), and GPC3 expression was closely related with HBsAg positivity, but not with sex, HCC differentiation, age, serum AFP level, tumor size, and extrahepatic metastasis. The high expression of GPC3 in HCC is similar to previous studies [9], [10], [11], [12], [13], [14], [15], [16], [17], [18], and further supports the diagnostic value of GPC3 for HCC. However, some authors have demonstrated positive staining in benign liver nodules ranging from none [25] to 7% to 22% of HGDN [6], [10], [26], [27] and to 75% of HGDN and 25% of LGDN [19]. Other reports have found variable degrees of positivity in benign cirrhotic nodules, although the staining is usually described as weak and focal [19]. Moreover, some authors considered that the transition from premalignant lesions to small HCC is associated with a sharp increase in GPC3 expression in a majority of cases. Simultaneously, the occurrence of small focal positivity for GPC3 in liver cirrhosis indicated strongly HCC regardless of the percentage of positive cells for GPC3 [10]. Thus, GPC3 positive liver nodules may have important value for monitoring the occurrence and early diagnosis of HCC.

Like other cancers, HCC is also characterized by an obvious multistage process of tumor progression [5], [28], [29] based on histopathological and molecular studies. The greatest number of HCC occurs mainly in liver cirrhosis caused by hepatitis B and C infection. Precancerous lesions known as DF and DN also appear in the chronically diseased liver, especially cirrhotic liver. Thus, it is of clinical importance to investigate the expression of GPC3 in precancerous lesion, the relationship between GPC3-positive nodules and HCC, and whether there is a genetic link between GPC3- positive nodules and HCC.

Monoclonality is an important characteristic of most tumors. In our previous studies, we ever microdissected 93 nodular lesions from 9 liver cirrhosis tissues from female patients, observed their histopathological features, and examined their clonality status. The results showed that loss of X chromosomal inactivation mosaicism was detected in 3 large regenerative nodules and 12 HGDN, indicating their neoplastic nature. Among the 60 LGDN, 29 (48.3%) were shown to be monoclonal, while 4 clear-cell lesions and 14 regenerative nodules were found to be polyclonal. Thus, we concluded that some DN in the HBV-associated liver cirrhosis, particularly HGDN, was already neoplastic lesions. HGDN with SCC is a late event during DN progression and suggests a premalignant phenotype [21]. In this study, we firstly found that 19 out of 103 DN (18.4%), including 15 (50%, 15/30) HGDN and 4 (5.48%, 4/73) LGDN, were positive for GPC3. The ratio of HGDN expression was higher obviously than that of LGDN expression for GPC3. The results were similar to that reported in literatures. Therefore, GPC3 expression in HCC was congruent with the adjacent DN in the same patient. In other words, GPC3 was detected in the adjacent HCC if it was also detected in the DN. On the contrary, it was not necessary to express GPC3 in adjacent DN if it was positive for HCC. Thus, we concluded that GPC3-positive DN, especially GPC3-positive HGDN, was closely related to HCC. Second, we found that all 15 GPC3-positive DN and 28 GPC3-negative DN from females were monoclonal. Moreover, all 24 HGDN from females were monoclonal regardless whether they expressed GPC3. These results support further that there is a close relationship between GPC3-positive DN in cirrhotic hepatic tissues and HCC though it remains controversial to use GPC3 to screen for premalignant hepatic lesions [13], [26], [30]. At least we can consider GPC3-positive DN, especially HGDN, to be late precancerous lesion of HCC, making it possible to monitor for the occurrence of HCC. Of course, additional studies must be performed to investigate the potential role of GPC3 during liver cirrhosis transformation to HCC. In addition, we found that 3 GPC3-positive LGDN with monoclonal hyperplasia were from two patients without HGDN. Moreover, the 3 GPC3-positive LGDN located in adjacent HCC. This indicated that part of GPC3-positive LGDN might directly progress into HCC. Interestingly, we observed that only several cells were positive for GPC3 in a few LGDN. However, the clonal assay revealed that these DN were also monoclonal. The most likely explanation for this is that these GPC3-positive cells are the ones that will eventually transform to HCC during hepatocarcinogenesis. Of course, further studies are required to determine the pathogenesis, significance, and other potential diagnostic uses of GPC3 in non-neoplastic liver disease.

Of course, clonal assays based on X-chromosome inactivation mosaicism and polymorphism of AR gene used in this study has its limitations. Specifically, it can only be used to analyze samples from female individuals. Molecular genetic studies have revealed that the occurrence of malignant tumors is related to the activation of oncogenes and inactivation of tumor suppressor genes (TSG). An important form of TSG inactivation is the mutation of one allele and the loss of heterozygosity (LOH) of the other allele. To further expand our analyses and confirm further our conclusions, we selected 11 microsatellite markers to assess the chromosomal LOH of 81 DN from females and 22 DN from males. The results demonstrated that a high frequency of LOH was detected at loci D6S1008, D8S262 and D11S1301, respectively. Moreover, the LOH frequency was significantly higher in GPC3-positive DN than in GPC3-negative DN at any loci. This indicated that genetic changes occurred in these GPC3-positive DN, further supporting that they represented the premalignant lesions of HCC. Moreover, the ratio of HGDN was higher than LGDN once there was chromosomal LOH at any loci. This indicated that there was a close relationship between HGDN and its adjacent HCC. Thus, we concluded that GPC3-positive DN, especially GPC3-positive HGDN, might be considered as a biomarker for the early diagnosis and detection of HCC. Moreover, the tumor suppressor gene adjacent to D6S1008, D8S262 and D11S1301 may be related to the occurrence of early HCC.

In conclusions, we concluded that GPC3-positive DN, especially GPC3-positive HGDN, was really a late premalignant lesion of HCC.
